# In situ-forming manganese-curcumin nanozyme film promotes diabetic wound healing via photothermal antibacterial and anti-inflammatory actions

**DOI:** 10.1016/j.mtbio.2026.103759

**Published:** 2026-10-19

**Authors:** Yuxin Wang, Long Wang, Weifeng Yang, Bingjie Sun, Xinyao Huang, Xiaohong Wang, Meiyu Zhang, Xinyu Wang, Jing Tu

**Affiliations:** a State Key Laboratory of Advanced Technology for Material Synthesis and Processing, Wuhan University of Technology, Wuhan, 430070, China; b Experimental Research Center, China Academy of Chinese Medical Sciences, Beijing, 100700, China

**Keywords:** Curcumin, Nanozyme, Prussian blue, ROS scavenging, Diabetic wound healing

## Abstract

Oxidative stress, bacterial infection, and persistent inflammatory responses are key inhibitory factors for chronic wound healing, yet most conventional dressings fail to simultaneously address these pathological barriers. This study presents a multifunctional composite film incorporating manganese-curcumin (Mn-Cur) nanozymes prepared via a facile, carrier-free synthesis. These nanozymes exhibit dual superoxide dismutase (SOD)- and catalase (CAT)-mimetic activities, enabling efficient elimination of superoxide anion radicals (•O_2_^−^) and hydrogen peroxide (H_2_O_2_). A rapid Prussian blue (PB) coating is introduced onto the Mn-Cur surface to enhance biocompatibility and confer photothermal antibacterial capability, forming Mn-Cur@PB (MC@PB) nanocomposites. The engineered nanozymes efficiently scavenge excess reactive oxygen species (ROS), exert potent antibacterial activity, and attenuate pro-inflammatory macrophage activation while inducing an immunoregulatory phenotype. The MC@PB nanocomposites are subsequently incorporated into a silk fibroin (SF)/polyvinyl butyral (PVB) matrix and cast into an in situ-forming wound dressing (denoted SPMP), bridging nanomaterial design and practical wound management. *In vivo* evaluation using infected diabetic wound models confirms that, under near-infrared (NIR) irradiation, the SPMP dressing effectively eliminates bacteria, alleviates inflammation, facilitates collagen deposition and angiogenesis, and accelerates wound closure. This multifunctional SPMP film represents a promising candidate for clinical chronic wound treatment.

## Highlights

• A carrier-free Mn-Cur nanozyme with SOD/CAT-mimicking activities is developed.
• Mn-Cur@PB integrates dual ROS-scavenging and photothermal sterilization capabilities.
• The PB coating confers photothermal antibacterial capability and biocompatibility.
• SPMP + NIR eliminates infection, alleviates inflammation, and promotes angiogenesis.
• The SPMP achieves significantly accelerated healing in diabetic infected wounds.

## Graphical abstract

MC@PB nanozymes with cascade ROS-scavenging, photothermal bactericidal, and immunoregulatory activities are formulated into a breathable in situ-forming film (SPMP) that reduces bacterial burden, alleviates inflammation, and promotes wound healing in diabetic wounds.

**Figure:**
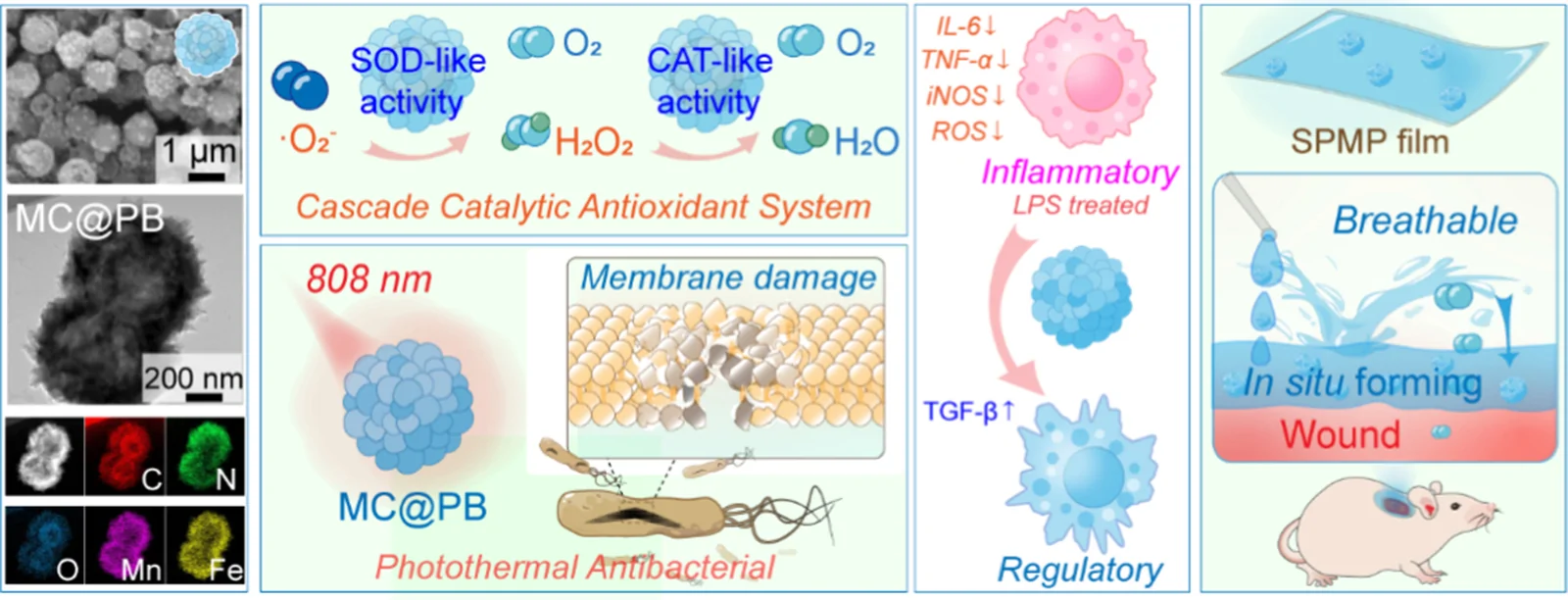

## 1 Introduction

Chronic wound healing remains a formidable clinical challenge, as persistent infection, excessive oxidative stress, and prolonged inflammation create a self-perpetuating cycle that stalls tissue repair [1], [2], [3]. Among hospitalized patients with chronic wounds, 50–60% present with active infection, while redox imbalance and vascular compromise further foster a pro-inflammatory microenvironment rich in reactive oxygen species (ROS) [4], [5]. This pathological milieu impairs tissue regeneration and impedes the critical transition from the inflammatory to the proliferative phase [6]. Current standard of care primarily relies on surgical debridement combined with antibiotics or skin grafting; however, these approaches are procedurally complex, economically burdensome, and fail to simultaneously address the infectious, oxidative, and inflammatory barriers [7], [8], [9], [10]. Therefore, developing a multifunctional wound dressing that integrates antibacterial, antioxidant, and anti-inflammatory capabilities into a single, easy-to-apply platform represents an urgent clinical need for advancing chronic wound management [8], [11] (see Scheme 1)

**Scheme 1:**
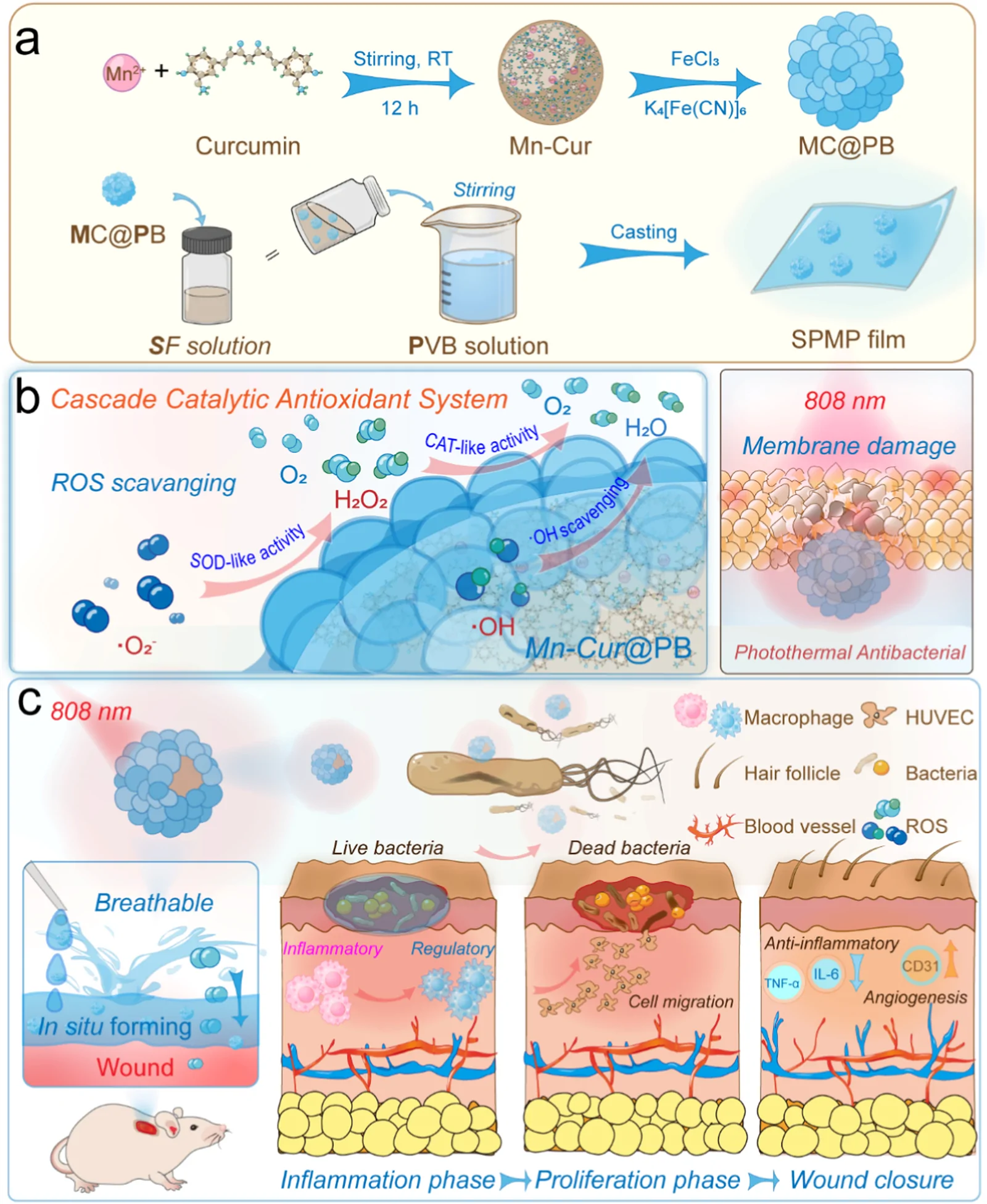
Schematic illustration of the MC@PB nanozyme-integrated multifunctional film for promoting diabetic wound healing by alleviating oxidative stress and inflammation. (a) Fabrication process of MC@PB nanozymes and the multifunctional film. (b) Mechanisms of ROS scavenging and antibacterial activity of the nanozymes. (c) Mechanisms of the multifunctional film in diabetic wound treatment.

To address this need, the rapid advancement of nanotechnology and a growing understanding of catalytic mechanisms have enabled the rational design of nanozymes with multiple enzyme-like activities [7], [12], [13]. These artificial enzymes offer distinct advantages over natural enzymes, including ease of preparation, catalytic diversity, tunable performance, and high stability, all of which facilitate large-scale production and clinical translation [14]. Among various nanozymes, those assembled from small-molecule ligands and metal ions via coordination interactions, such as metal-organic frameworks (MOFs) and metal-phenolic networks (MPNs), have attracted considerable attention [15], [16]. Polyphenols, including tannic acid, epigallocatechin gallate, gallic acid, and curcumin, serve as excellent ligands due to their abundant chelating groups. These polyphenol-metal nanozymes self-assemble via metal coordination, π-π stacking, and hydrophobic interactions [17]. The metal centers typically provide superoxide dismutase (SOD)-like and catalase (CAT)-like activities for scavenging ROS, while the phenolic moieties contribute to direct radical quenching and bacterial membrane disruption [18]. Consequently, polyphenol-metal nanozymes hold significant potential for regulating oxidative stress and infection at chronic wound sites [19]. For instance, Huang et al. reported a caffeic acid-vanadium nanozyme (CA-V) with dual SOD/CAT-mimetic activities to scavenge ROS/RNS, downregulate TNF-α and IL-17 inflammatory pathways, and promote angiogenesis and collagen remodeling, thereby accelerating acute wound healing [20]. Similarly, Shi et al. engineered a tannic acid-Fe^3+^/glucose oxidase system (LGTF) to achieve glucose-responsive synergistic disinfection of hyperglycemic wounds through in situ generation of ROS and NO, while self-amplifying its catalytic activity by lowering local pH, a strategy that exploits ROS generation for disinfection rather than scavenging [21], [22]. These examples underscore the versatility of polyphenol-metal platforms in modulating the wound microenvironment.

Curcumin is a natural polyphenol with broad sources, low cost, and easy accessibility. It exhibits excellent antioxidant, antibacterial, and anti-inflammatory activities, along with good biocompatibility. Its unique β-diketone moiety and phenolic hydroxyl groups enable efficient chelation of various metal ions including Zn^2+^, Cu^2+^, Fe^3+^, and Mn^2+^, demonstrating substantial potential for nanozyme synthesis [23]. However, most reported studies have focused on curcumin coordinated with Zn^2+^ to form nanoparticles [24], [25], [26], [27], [28], [29]. Their synthesis typically requires heating, and the resulting nanoparticles often lack inherent enzyme-mimicking activity. Their ability to scavenge ROS primarily stems from the phenolic hydroxyl groups of curcumin itself. In contrast, nanozymes possessing both SOD-like and CAT-like activities can efficiently convert excess superoxide anion and hydrogen peroxide at diabetic wound sites into water and oxygen through a cascade catalytic reaction. This capability can effectively alleviate oxidative stress and promote the transition of wounds from the inflammatory phase to the proliferative phase [8].

To deliver such nanozymes to chronic wounds, an appropriate dressing platform is required. Compared with traditional dressings (e.g., gauze, bandages), in situ-forming dressings (e.g., hydrogels, films) can adapt to irregular wound shapes, offer excellent breathability and water resistance, and serve as ideal carriers for various drugs and nanoparticles [11]. Silk fibroin is a natural polymer that can be extracted via a simple process. Its molecular weight can be controlled by adjusting the processing methods, thereby regulating the mechanical properties of the resulting materials. In addition, its excellent biocompatibility has garnered widespread attention [30]. When combined with polyvinyl butyral, silk fibroin can rapidly form a film at the wound site, covering the injury to prevent secondary damage and pathogen invasion [11].

In this study, amorphous Mn^2+^-curcumin coordinated nanozymes (denoted Mn-Cur) were fabricated at room temperature via sequential coordination and self-assembly. These Mn-Cur nanozymes exhibit excellent SOD- and CAT-like activities, enabling the cascade elimination of •O_2_^−^ and H_2_O_2_, while the uncoordinated phenolic hydroxyl groups of curcumin within Mn-Cur further scavenge •OH. To further enhance the therapeutic functionality, Prussian blue (PB) was decorated onto the surface of Mn-Cur, obtaining Mn-Cur@PB (MC@PB) nanocomposites. This PB coating not only modulates the valence state of Mn, thereby tuning its SOD- and CAT-like enzymatic activities, but also endows the system with photothermal conversion capability and improved biocompatibility. The resulting MC@PB nanozymes effectively eliminate *S. aureus* and *E. coli* under 808 nm near-infrared (NIR) irradiation. In addition, they attenuate pro-inflammatory macrophage activation and induce an immunoregulatory phenotype, thereby alleviating inflammatory responses. To meet the practical demands of diabetic wound treatment, MC@PB was incorporated into an SF-PVB solution, which can be applied onto the wound site to form a multifunctional film (denoted SPMP) in situ, adapting to irregular wound shapes. In a full-thickness skin defect model in diabetic mice, the SPMP dressing effectively eliminates pathogens, alleviates inflammation, and accelerates diabetic wound healing.

## 2 Results and discussion

### 2.1 Synthesis and characterization of Mn-Cur and MC@PBs nanozymes

Mn-Cur nanozymes were fabricated at room temperature via coordination-driven self-assembly between curcumin and Mn(CH_3_COO)_2_ in DMF/ethanol (Fig. 1a). The effects of the dilution method and the direct mixing method on the morphology of Mn-Cur were further compared (Fig. S1). In both cases, obvious precipitates formed within approximately 4 h, and the resulting Mn-Cur particles were spherical. TEM images revealed that, for the dilution method, the particle size gradually increased with reaction time, with values of 314.8 ± 75.3 nm at 4 h, 462.7 ± 91.6 nm at 6 h, and 569.4 ± 117.9 nm at 24 h. In contrast, the direct mixing method yielded slightly smaller particles, with sizes of 236.1 ± 70.0 nm at 4 h, 310.1 ± 90.0 nm at 6 h, and 436.7 ± 109.7 nm at 24 h, which was attributed to its faster nucleation rate [25]. Based on these results, the dilution method was selected for all subsequent experiments.

**Fig. 1:**
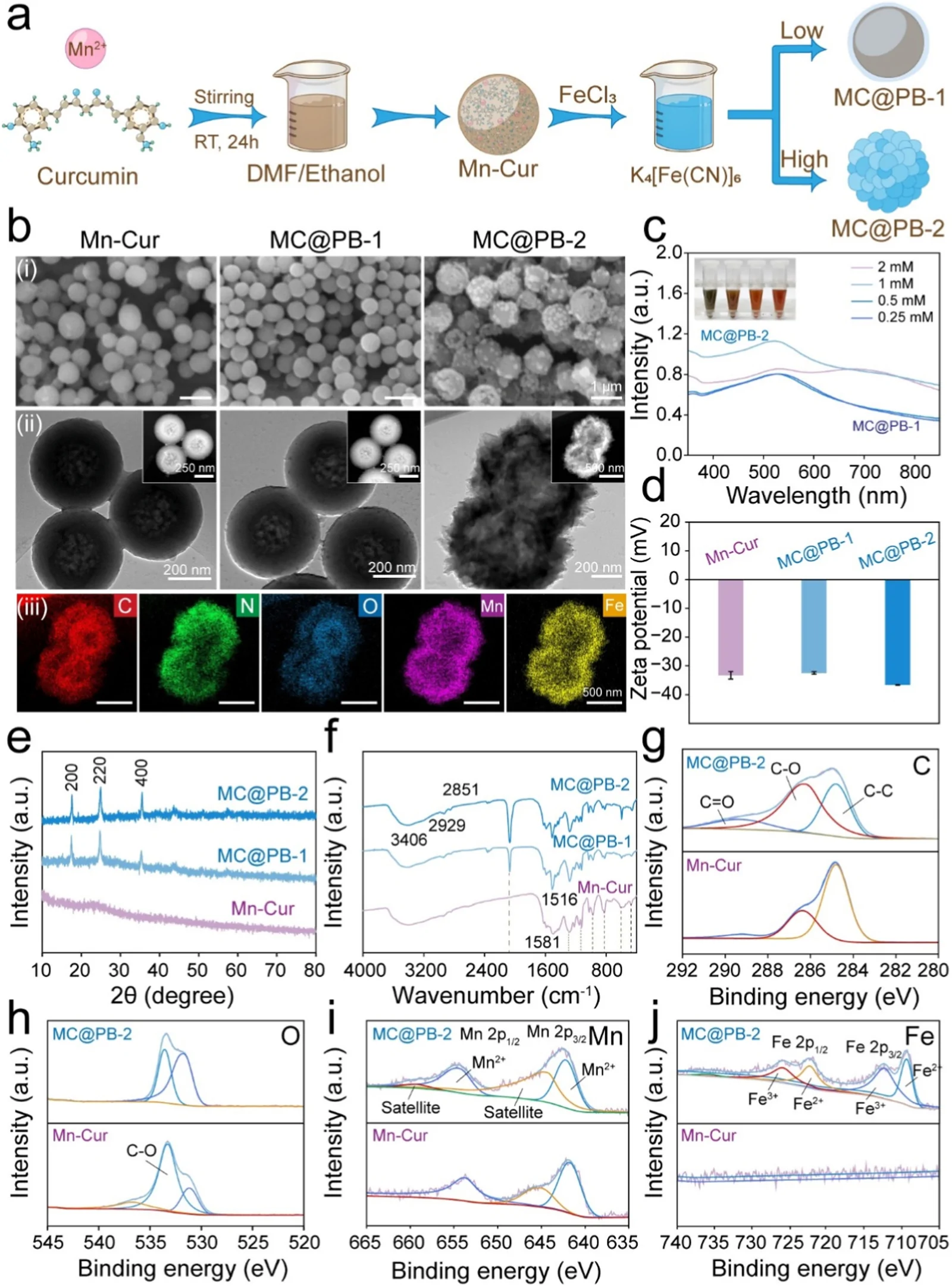
(a) Synthetic routes of Mn-Cur, MC@PB-1 and MC@PB-2. (b) SEM, TEM, HAADF (insert) and EDS mapping images of Mn-Cur, MC@PB-1 and MC@PB-2. (c) UV–vis absorption spectra of MC@PBs synthesized with FeCl_3_ and K_4_[Fe(CN)_6_] at different equimolar concentrations (0.25, 0.5, 1, and 2 mM). (d) Zeta potential (in deionized water), (e) XRD patterns, and (f) FTIR spectra of Mn-Cur, MC@PB-1 and MC@PB-2. High-resolution XPS spectra of (g) C 1s, (h) O 1s, (i) Mn 2p and (j) Fe 2p of Mn-Cur, MC@PB-1, and MC@PB-2.

Prussian blue (PB) was deposited onto the surface of Mn-Cur via the slow addition of FeCl_3_ aqueous solution to the Mn-Cur dispersion containing K_4_[Fe(CN)_6_]. By varying the precursor concentrations, a series of MC@PB hybrids was prepared (Fig. 1a and S2). SEM and TEM imaging revealed that the Mn-Cur particles (collected after 12 h of aging) were spherical with smooth surfaces and a mean diameter of 467.95 ± 68.89 nm, whereas the MC@PB hybrids exhibited a distinct core-shell architecture. At precursor concentrations of 0.25 mM, the nanozyme surface became slightly roughened and the particle size increased to 575.48 ± 98.47 nm. When the concentrations were further increased to 1 mM, a dense PB layer completely coated the Mn-Cur core, resulting in a particle size of 670.90 ± 135.72 nm. The shell thickness of MC@PB increased progressively with higher precursor concentrations (Fig. 1b i, ii, and S2).

EDS elemental mapping confirmed the homogeneous distribution of C, O, and Mn throughout both Mn-Cur and MC@PB-1, with N and Fe uniformly distributed over the surface of MC@PB-1 (Fig. S3). In contrast, MC@PB-2 displayed a clear elemental segregation, wherein C and O were enriched in the core, while N and Fe were predominantly confined to the shell layer. Interestingly, Mn also showed a tendency to migrate toward the shell region (Fig. 1b iii). UV-vis absorption spectra indicated that MC@PBs exhibited an absorption peak at approximately 540 nm, with a concomitant increase in absorbance near 750 nm as the PB content increased (Fig. 1c). Zeta potential measurements showed that all nanozymes possessed a negative surface charge, with ζ-potential values ranging from −30 to −40 mV (Fig. 1d). XRD patterns suggested an amorphous structure for Mn-Cur, whereas the surface-deposited PB displayed a crystalline nature, with characteristic diffraction peaks at 17.4°, 24.7°, and 35.2°, corresponding to the (200), (220), and (400) planes, respectively (Fig. 1e) [31].

FTIR spectroscopy was employed to characterize the functional groups of the three nanozymes (Fig. 1f). The absorption band at 3406 cm^−1^ was attributed to O-H stretching vibrations, while the peaks at 1620, 1581, and 1516 cm^−1^ corresponded to C=C stretching. The peak at 1407 cm^−1^ arose from C-H bending within the benzene ring and saturated hydrocarbon groups. Multiple C-O stretching vibrations were observed at 1279, 1225, 1166, 1130, and 1030 cm^−1^, and the bands at 980 and 821 cm^−1^ were characteristic of = C-H out-of-plane wagging. The peak at 476 cm^−1^, attributed to Mn-O stretching, confirmed the successful formation of Mn-Cur [32], [33]. Furthermore, the distinct absorption at 2072 cm^−1^, assigned to the C ≡ N stretching vibration, verified the successful modification with PB [31].

XPS was further performed to probe the surface chemical states of Mn-Cur and MC@PB-2 (Fig. 1g–j). The surface atomic percentages of Mn, C, and O in Mn-Cur were determined to be 2.0%, 80.2%, and 16.8%, respectively (Fig. S4). Notably, PB modification did not reduce the surface Mn content; instead, the surface Mn content slightly increased to 2.8%, further supporting the migration of Mn toward the shell observed in the elemental mapping. Both Mn-Cur and MC@PB-2 exhibited distinct peaks corresponding to the C 1s, O 1s, and Mn 2p core levels. The C 1s spectrum was deconvoluted into C-C (284.6 eV) and C-O/C=O (286.1 eV) components, while the O 1s spectrum displayed peaks at 533.3 eV (C-O) and 532.1 eV (C=O). The Mn 2p spectrum showed peaks at 642.0 eV (Mn(II) 2p_3_/_2_) and 653.9 eV (Mn(II) 2p_1_/_2_). MC@PB-2 additionally revealed Fe 2p peaks (Fig. 1j), with binding energies at 709.3 and 721.6 eV assigned to Fe(II) 2p_3_/_2_ and Fe(II) 2p_1_/_2_, respectively, and at 711.2 and 724.2 eV assigned to Fe(III) 2p_3_/_2_ and Fe(III) 2p_1_/_2_, respectively. Notably, a characteristic peak corresponding to Mn(III) emerged at approximately 643.0 eV after PB coating, suggesting an increased proportion of Mn(III) species [34], [35].

### 2.2 Antioxidant capacity of Mn-Cur, MC@PB-1 and MC@PB-2

The antioxidant performance of Mn-Cur, MC@PB-1, and MC@PB-2 was systematically evaluated by assessing their scavenging abilities toward H_2_O_2_, •O_2_^−^, •OH, ABTS•^+^, and DPPH• (Fig. 2; detailed spectra are provided in Fig. S5). The proposed antioxidant mechanism of MC@PB-2 is depicted in Fig. 2a, in which nanozymes alleviate hypoxia and intracellular oxidative stress through a cascade enzymatic reaction: SOD-like dismutation of •O_2_^−^ to H_2_O_2_ and O_2_, followed by CAT-like decomposition of H_2_O_2_ to H_2_O and O_2_. Meanwhile, the phenolic hydroxyl groups on the curcumin surface contribute to •OH scavenging, achieving synergistic antioxidant protection.

**Fig. 2:**
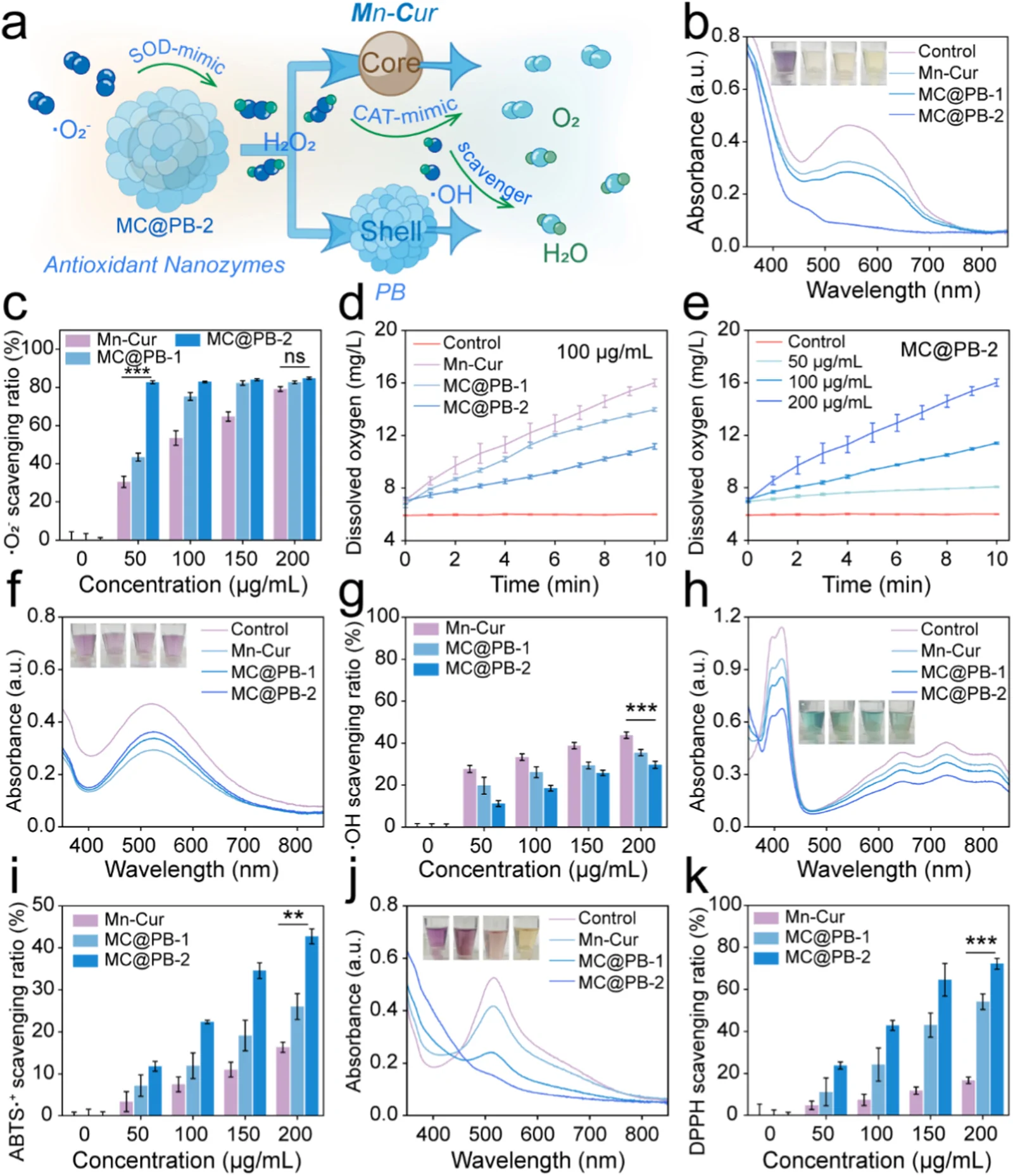
(a) Schematic illustration of the ROS scavenging mechanism of MC@PB. (b) NBT photoreduction inhibition of Mn-Cur, MC@PB-1, and MC@PB-2 (50 μg/mL) and (c) •O_2_^−^ scavenging ratio at different concentrations (0–200 μg/mL). (d) CAT-mimetic activity of Mn-Cur, MC@PB-1, and MC@PB-2 at 100 μg/mL (e) CAT-mimetic activity of MC@PB at various concentrations (0–200 μg/mL). (f) UV–vis absorption spectra of 2OH-SA after treatment with Mn-Cur, MC@PB-1, and MC@PB-2 (200 μg/mL) and (g) corresponding •OH scavenging ratios at different concentrations (0–200 μg/mL). (h) UV–vis absorption spectra of ABTS•^+^ after treatment with Mn-Cur, MC@PB-1, and MC@PB-2 (200 μg/mL) and (i) corresponding scavenging ratios at different concentrations (0–200 μg/mL). (j) UV–vis absorption spectra of DPPH• after treatment with Mn-Cur, MC@PB-1, and MC@PB-2 (200 μg/mL) and (k) corresponding scavenging ratios at different concentrations (0–200 μg/mL). Data are presented as mean ± SD (n = 3). *p < 0.05, **p < 0.01, ***p < 0.001; ns, not significant.

As presented in Fig. 2b, the •O_2_^−^ scavenging ratios followed the trend: MC@PB-2 > MC@PB-1 > Mn-Cur. Under identical experimental conditions, MC@PB-2 exhibited an •O_2_^−^ scavenging ratio of 82.62 ± 0.79% at 50 μg/mL, whereas Mn-Cur achieved only 79.15 ± 1.22% even at a much higher concentration of 200 μg/mL (Fig. 2c). To evaluate CAT-like activity, O_2_ production was monitored. As shown in Fig. 2d and e, all three nanozymes effectively decomposed H_2_O_2_ into O_2_ and H_2_O, with catalytic efficiency following the order: Mn-Cur > MC@PB-1 > MC@PB-2. MC@PB-2 also exhibited CAT activity in a concentration-dependent manner. These results suggest that PB surface decoration reduced CAT-like activity while enhancing SOD-like activity, which may be associated with the change in Mn valence distribution on the nanozyme surface. The SOD-like activity of Mn-based nanozymes involves the Mn^3+^/Mn^2+^ redox cycle, and the presence of Mn(III) may favor this cycle, consistent with the enhanced SOD-like activity. In contrast, CAT-like activity involves Mn^2+^-dependent H_2_O_2_ activation, and the formation of mixed Mn(II)/Mn(III) species may alter the Mn^2+^-dependent H_2_O_2_ activation process, consistent with the reduced CAT-like activity. In addition, the PB shell may influence the net CAT-like activity through multiple factors, including possible Fe-mediated H_2_O_2_ catalysis and steric limitation of H_2_O_2_ access to Mn active sites.

Furthermore, PB modification decreased the •OH scavenging ability, with scavenging ratios of 43.81 ± 1.53% (Mn-Cur), 35.51 ± 1.46% (MC@PB-1), and 29.71 ± 1.65% (MC@PB-2) at 200 μg/mL (Fig. 2f and g). This decline likely arises from the coordination between curcumin phenolic hydroxyls and Fe^3+^/Fe^2+^ ions in the PB lattice, which reduces the availability of active hydrogen donors essential for •OH scavenging. Additionally, the electron-withdrawing Fe-CN network further diminishes the hydrogen-donating capacity of phenolic oxygens. Although PB itself exhibits intrinsic •OH scavenging via Fe^2+^/Fe^3+^ redox cycling, the net activity of the hybrid system is dominated by surface-exposed curcumin, whose accessibility to radicals is restricted by interfacial coordination.

ABTS•^+^ and DPPH• scavenging activities were also investigated. Mn-Cur showed limited efficiency toward both ABTS•^+^ (16.35 ± 1.20%) and DPPH• (16.70 ± 1.55%) at 200 μg/mL, likely due to coordination between Mn^2+^ and the phenolic hydroxyl groups of curcumin, which may hinder radical access. Notably, PB modification substantially improved both scavenging activities. For ABTS•^+^, scavenging ratios reached 25.99 ± 3.04% (MC@PB-1) and 42.77 ± 1.78% (MC@PB-2) (Fig. 2h and i). More pronounced enhancements were observed for DPPH•, with ratios of 54.08 ± 3.67% (MC@PB-1) and 72.23 ± 2.56% (MC@PB-2) (Fig. 2j and k).

### 2.3 Antibacterial activity of Mn-Cur, MC@PB-1, and MC@PB-2

Photothermal therapy (PTT) is a promising technique which can disrupt the intact structure of bacteria by non-invasively generating high temperature within their bioactive substrates, such as proteins and nucleic acids, making it easier for antibacterial agents to penetrate and eradicate the protected bacteria [36]. Diabetic wounds are highly susceptible to bacterial infection due to their complex pathological microenvironment [1]. The antibacterial activity of the nanozymes was evaluated using the spread plate method against *S. aureus* and *E. coli*. Under 808 nm NIR irradiation, the nanozymes were capable of killing bacteria by disrupting cell membrane integrity (Fig. 3a).

**Fig. 3:**
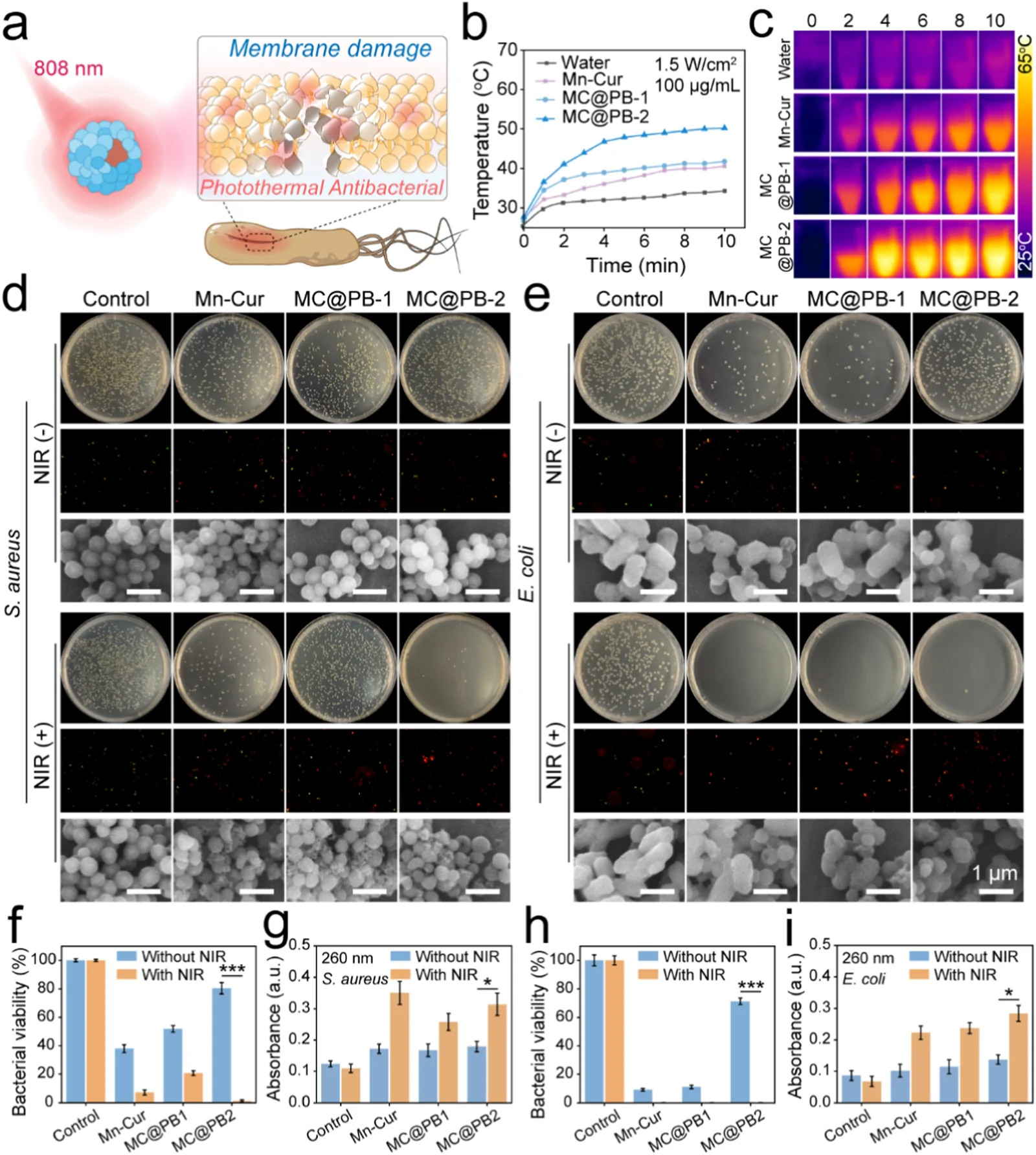
(a) Schematic illustration of the antibacterial mechanism of MC@PB. (b) Photothermal heating curves of Mn-Cur, MC@PB-1, and MC@PB-2 (100 μg/mL) under NIR irradiation (808 nm, 1.5 W/cm^2^) and (c) corresponding real-time infrared thermal images. (d) Photographs of *S. aureus* and (e) *E. coli* colonies on agar plates; corresponding live/dead fluorescence staining images (green: live, red: dead) and SEM images after different treatments. (f) Antibacterial ratios and (g) absorbance at 260 nm of the supernatant against *S. aureus* after different treatments. (h) Antibacterial ratios and (i) absorbance at 260 nm of the supernatant against *E. coli* after different treatment. Data are presented as mean ± SD (n = 3). *p < 0.05, **p < 0.01, ***p < 0.001; ns, not significant. (For interpretation of the references to color in this figure legend, the reader is referred to the Web version of this article.)

The decoration of PB enhanced the nanozyme's photothermal performance, enabling MC@PB-2 to heat its dispersion (100 μg/mL) to 50.2 °C in 10 min (the power of NIR was 1.5 W/cm^2^). Under identical experimental conditions, other nanozymes were incapable of raising the temperature of their dispersion to 50 °C, with Mn-Cur reaching only 40.6 °C and MC@PB-1 reaching 41.8 °C (Fig. 3b and c). In addition, the photothermal performance of MC@PB-2 under various conditions is presented in Fig. S6, and its photothermal conversion efficiency was determined to be 10.54%.

Colony counting results showed that, without NIR irradiation, MC@PB-2 exhibited weaker antibacterial activity than Mn-Cur and MC@PB-1, likely attributable to the PB shell restricting direct contact between the Mn-Cur core and bacteria (Fig. 3d, e and S7). Specifically, after treatment with Mn-Cur (200 μg/mL) in the absence of NIR, bacterial viability was 37.76 ± 2.76% for *S. aureus* and 9.29 ± 0.87% for *E. coli*. In contrast, MC@PB-2 without NIR yielded survival rates of 80.49 ± 3.93% (*S. aureus*) and 71.40 ± 2.31% (*E. coli*). However, upon NIR irradiation, bacterial viability decreased markedly after incubation with MC@PB-2 (200 μg/mL), with survival rates decreasing to 3.93 ± 1.35% (*S. aureus*) and 0.08 ± 0.04% (*E. coli*) (Fig. 3f–h).

To further elucidate the antibacterial mechanism, live/dead staining (AO/EB) was performed on both *S. aureus* and *E. coli* under various treatment conditions. The results showed that bacteria incubated with MC@PB-2 under NIR irradiation exhibited more intense red fluorescence (indicative of dead bacteria) compared to those without NIR irradiation or the control groups. In addition, morphological changes were observed by SEM. Bacteria in the control group maintained smooth and intact surfaces, whereas those treated with MC@PB-2 displayed surface depression and wrinkling, with the most pronounced damage observed in the NIR-irradiated groups. Furthermore, an increased absorbance at 260 nm was detected in the supernatants of the NIR-irradiated MC@PB-2 groups (Fig. 3g–i), indicating that the photothermal effect disrupted the bacterial membrane and caused leakage of intracellular contents. Collectively, these results demonstrate that MC@PB-2 exerts potent bactericidal activity through a photothermal mechanism upon NIR irradiation.

### 2.4 Synthesis and characterization of SP and SPMP films

MC@PB-2-based composite films were constructed by incorporating SF and PVB, utilizing the anti-inflammatory and antioxidant properties of SF to form a rapidly in-situ film (SPMP) (Fig. 4a). Fig. 4b shows photographs of the precursor solutions of SP (SF/PVB without MC@PB-2) and SPMP, along with SEM images of the resulting films. Both SP and SPMP formed transparent films. Surface SEM images revealed rough morphologies, while cross-sectional views indicated a film thickness of approximately 50 μm, with MC@PB-2 particles embedded within the film matrix.

**Fig. 4:**
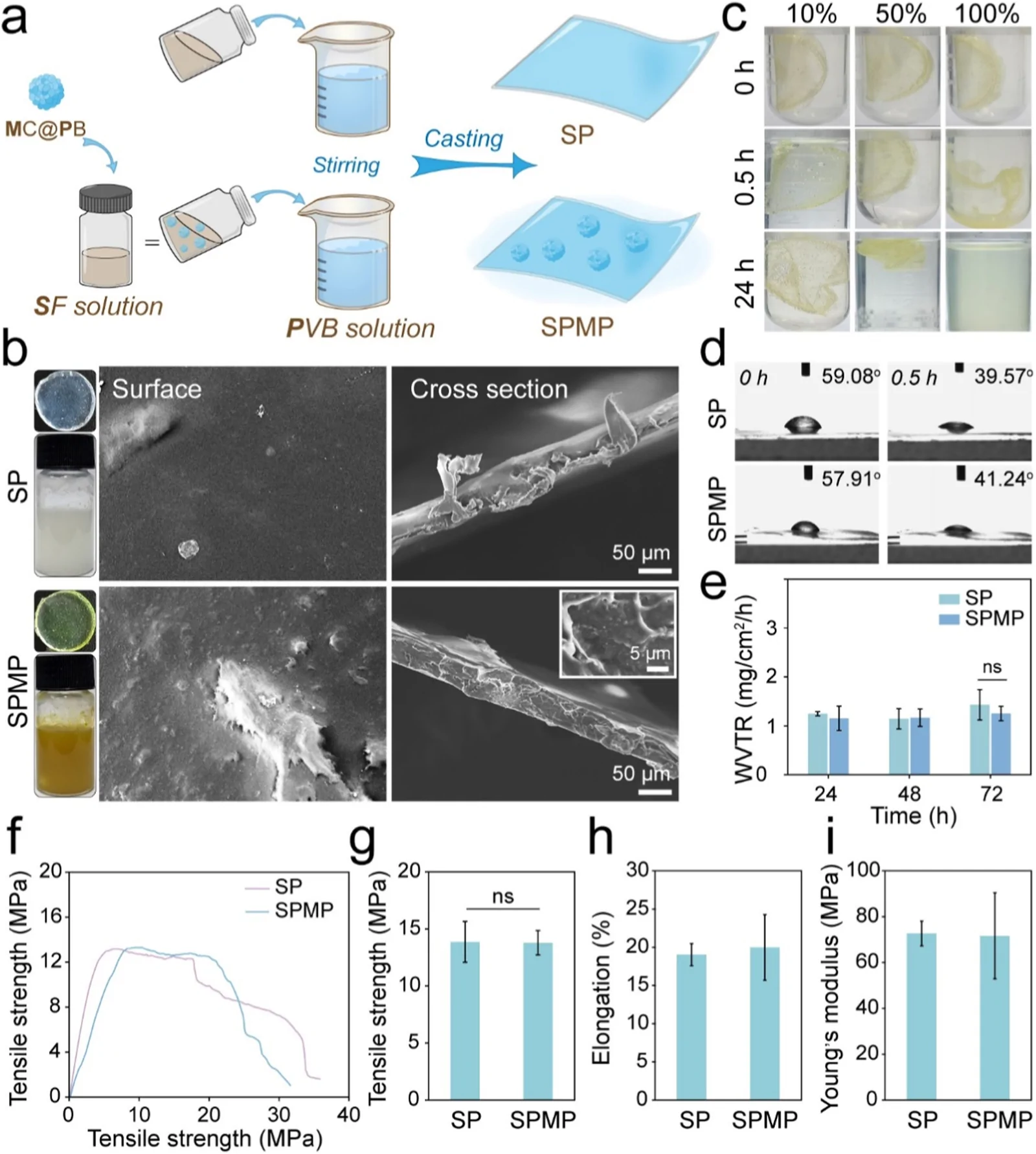
(a) Schematic illustration of the fabrication process of SP and SPMP films. (b) Optical photographs and SEM images of SP and SPMP. (c) Degradation behavior of SPMP in ethanol solutions at various concentrations (10%, 50%, and 100%). (d) Water contact angle measurements of SP and SPMP. (e) Water vapor transmission rate (WVTR) of SP and SPMP. (f) Tensile stress-strain curves of SP and SPMP. (g) Tensile strength, (h) elongation, and (i) Young's modulus of SP and SPMP. Data are presented as mean ± SD (n = 3).

The removability of SPMP was evaluated by immersing the films in 10%, 50%, and 100% ethanol solutions (Fig. 4c). SPMP dissolved more readily as the ethanol proportion increased. Notably, when immersed in anhydrous ethanol, SPMP completely dissolved within 24 h.

Film hydrophilicity was assessed by contact angle measurements (Fig. 4d). The contact angles of SP and SPMP were measured to be 59.08° ± 2.13° and 57.91° ± 1.54°, respectively. Furthermore, water droplets gradually infiltrated both films within 0.5 h, indicating good hydrophilicity, which facilitates cell adhesion, growth, and migration. Appropriate air permeability is beneficial for gas exchange at the wound site to maintain a moist environment [11], [37]. The water vapor transmission rates (WVTRs) of both SP and SPMP remained around 1.2 mg/(cm^2^•h) over three days, with no significant effect observed following MC@PB-2 incorporation (Fig. 4e).

The mechanical properties of the films were also evaluated. The tensile strengths of SP and SPMP were 13.87 ± 1.79 MPa and 13.78 ± 1.06 MPa, elongations at break were 19.03 ± 1.45% and 19.98 ± 3.29%, and Young's moduli were 72.73 ± 5.37 MPa and 71.67 ± 18.79 MPa, respectively (Fig. 4f–i). These results indicate that MC@PB-2 incorporation exerted a negligible influence on the mechanical integrity of the composite film. The comparable tensile strength and Young's modulus suggest uniform dispersion of MC@PB-2 within the SF/PVB matrix without introducing stress concentration. The maintained elongation at break further confirms that MC@PB-2 loading did not compromise film flexibility, which is essential for conformal wound contact and resistance to mechanical deformation during patient movement.

### 2.5 Cytocompatibility, anti-inflammatory activity, and cell migration of MC@PB-2 and SPMP

The cytocompatibility of the nanozymes and films was first evaluated on HUVECs using the MTT assay. As shown in Fig. 5a, PB modification enhanced the cytocompatibility of Mn-Cur. After 24 h of incubation at 100 μg/mL, all three nanozymes exhibited no obvious cytotoxicity, with cell viabilities of 92.78% ± 2.37% for Mn-Cur, 94.16% ± 1.22% for MC@PB-1, and 97.31% ± 2.31% for MC@PB-2. At 200 μg/mL, the viability of Mn-Cur dropped to 69.11% ± 1.73%, whereas MC@PB-1 and MC@PB-2 maintained substantially higher viabilities of 86.69% ± 1.68% and 99.14% ± 1.77%, respectively, indicating that PB surface coating alleviates the cytotoxicity of the bare nanozyme at elevated concentrations. When the incubation time was extended to 72 h, the nanozymes showed no significant effect on cell proliferation at concentrations below 50 μg/mL (Fig. 5b). In addition, no obvious hemolysis was observed for any of the three nanozymes at 200 μg/mL, indicating good hemocompatibility (Fig. 5c). SP and SPMP also exhibited good cytocompatibility, with HUVECs proliferating normally during 72 h of incubation with their extracts (Fig. S8).

**Fig. 5:**
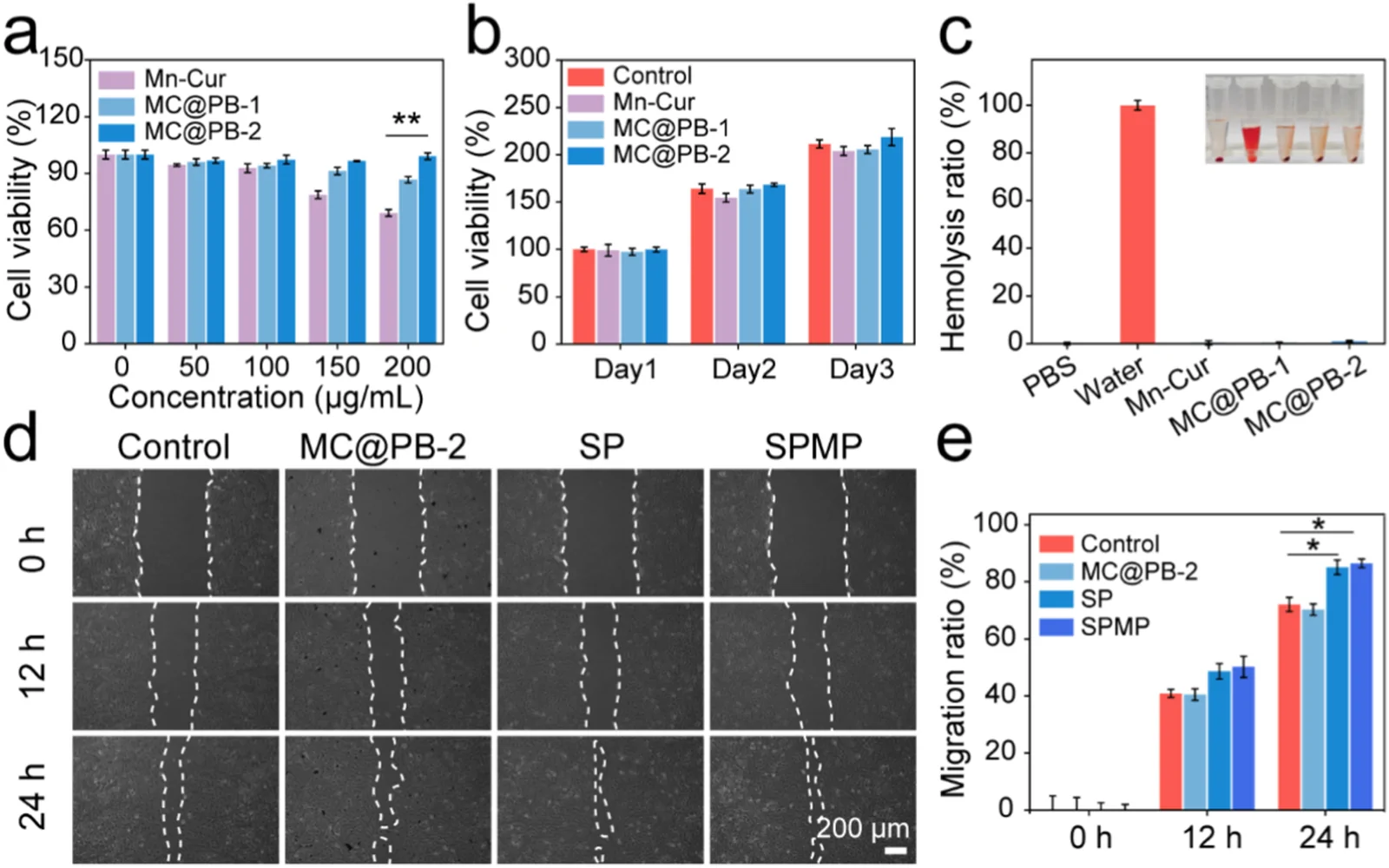
(a) Viability of HUVECs treated with various concentrations of MC@PB (0–200 μg/mL) for 24 h. (b) Viability of HUVECs treated with MC@PB (50 μg/mL) for 24, 48, and 72 h. (c) Hemolytic ratio of MC@PB (200 μg/mL) after incubation at 37 °C. (d) Representative images of HUVEC migration after different treatments and (e) corresponding migration ratios.

Scratch assay results (Fig. 5d and e) revealed that the migration rates of the SP (85.07 ± 2.54%) and SPMP (86.46 ± 1.52%) extract-treated groups were significantly higher than that of the control group (72.03 ± 0.49%) at 24 h, which may be attributed to the activation of the NF-κB signaling pathway by SF [38].

A moderate inflammatory response is beneficial for eliminating dead cells and pathogens, whereas excessive inflammation can delay the transition from the inflammatory phase to the proliferation phase, thereby impairing wound healing [5], [6]. An inflammatory model using LPS-stimulated macrophages was established to evaluate the anti-inflammatory effects of the three nanozymes. ELISA results (Fig. 6b and c) showed that LPS-stimulated macrophages secreted elevated levels of IL-6 and TNF-α, whereas subsequent incubation with the nanozymes significantly reduced the secretion of these pro-inflammatory cytokines compared with the positive control group. Furthermore, the fluorescence intensities of iNOS (a pro-inflammatory marker) and ROS in macrophages incubated with the nanozymes were significantly lower than those in the positive control group (Fig. 6d–f), supporting the anti-inflammatory activity of the nanozymes. We further examined the immunoregulatory markers IL-10, TGF-β, and Arg-1 (Fig. 6g–i). Among them, TGF-β secretion was significantly increased in the nanozyme-treated groups, whereas IL-10 and Arg-1 showed no significant changes. These results suggest that the nanozymes induce an immunoregulatory signal.

**Fig. 6:**
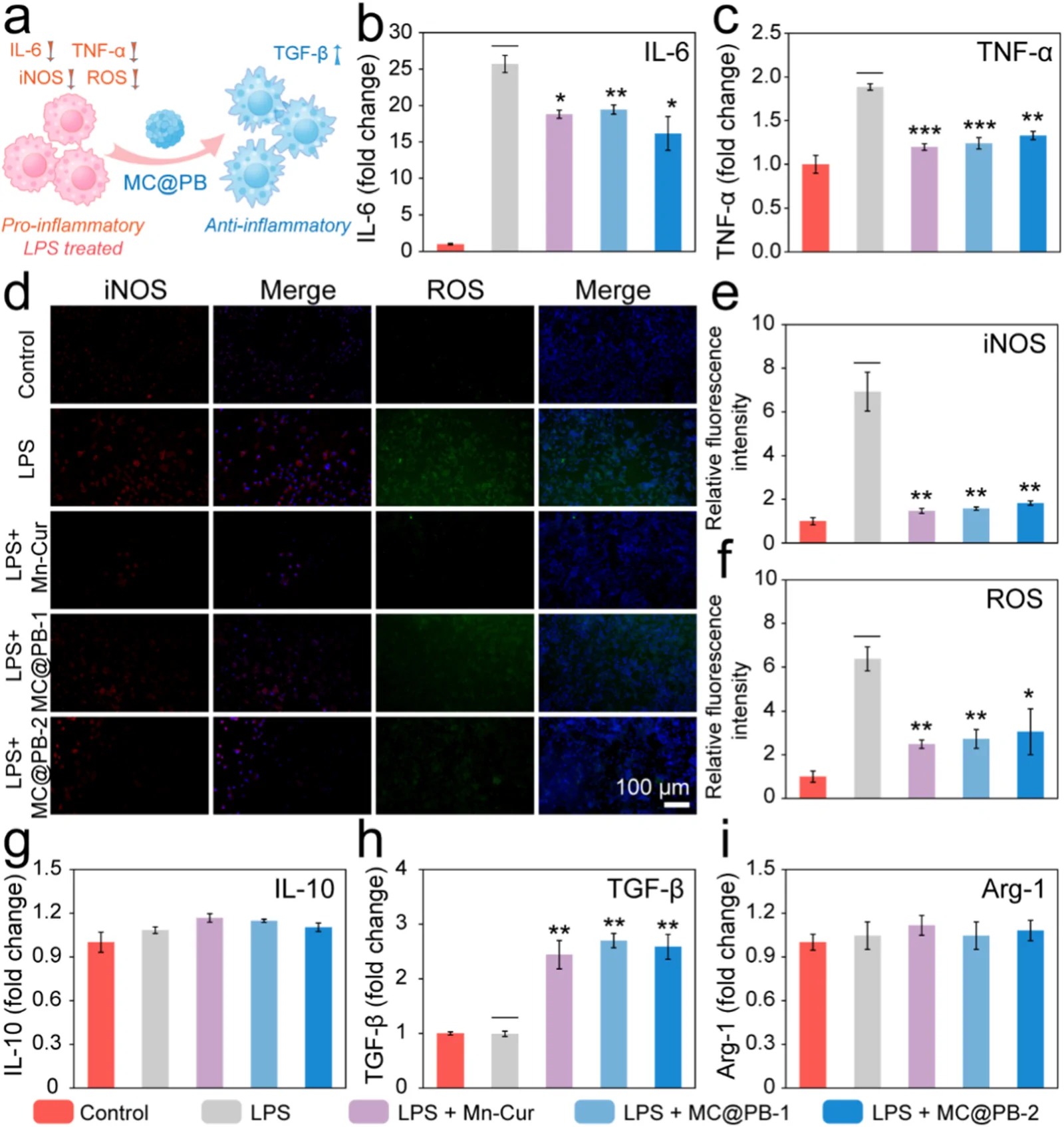
(a) Schematic illustration of MC@PB-2 attenuating pro-inflammatory macrophage activation and inducing an anti-inflammatory/immunoregulatory phenotype, characterized by decreased IL-6, TNF-α, iNOS, and ROS and increased TGF-β. Pro-inflammatory, LPS-treated M1-like macrophage; Anti-inflammatory, immunoregulatory phenotype. Secretion levels of (b) IL-6 and (c) TNF-α in Raw 264.7 macrophages after different treatments. (d) Immunofluorescence staining of iNOS (red), ROS (green), and nuclei (blue) in macrophages after different treatments; (e, f) corresponding relative fluorescence intensity of iNOS and ROS, respectively. Secretion levels of (g) IL-10, (h) TGF-β and (i) Arg-1 in Raw 264.7 macrophages after different treatments. TGF-β was significantly increased, whereas IL-10 and Arg-1 showed no significant changes. (For interpretation of the references to color in this figure legend, the reader is referred to the Web version of this article.)

### 2.6 *In vivo* diabetic wound healing performance in a mouse model

Based on the ROS-scavenging, antibacterial, and anti-inflammatory capabilities of MC@PB-2, combined with the in situ film-forming advantages of SPMP for practical wound care, an infected full-thickness skin defect model (wound diameter: 10 mm) was established in diabetic mice to evaluate the therapeutic potential of these materials for infected diabetic wounds (Fig. 7a). Compared with the control group (relative wound area: 38.60 ± 3.59% on day 14), all treatment groups exhibited smaller relative wound areas, with the SPMP + NIR group showing the most pronounced reduction (8.90 ± 2.74%). In the control group, wound suppuration occurred on day 7, indicating severe bacterial infection that significantly impeded the healing process. The MC@PB-2 + NIR group demonstrated relatively slower healing during the later stage (days 10–14), which may be attributed to the dilution and clearance of nanozymes by wound exudate, resulting in decreased local concentration at the wound site (Fig. 7b–e). Bacterial counts collected from wound tissues on day 5 (Fig. 7f) revealed that MC@PB-2 + NIR treatment significantly reduced bacterial burden compared to the control. Notably, the SP, SPMP, and SPMP + NIR groups exhibited even lower bacterial counts, likely due to the antibacterial effect of ethanol present in the precursor solution of the film-forming system. Throughout the 14-day treatment period, no significant changes in body weight were observed across all groups (Fig. 7g), suggesting negligible systemic toxicity of the treatments.

**Fig. 7:**
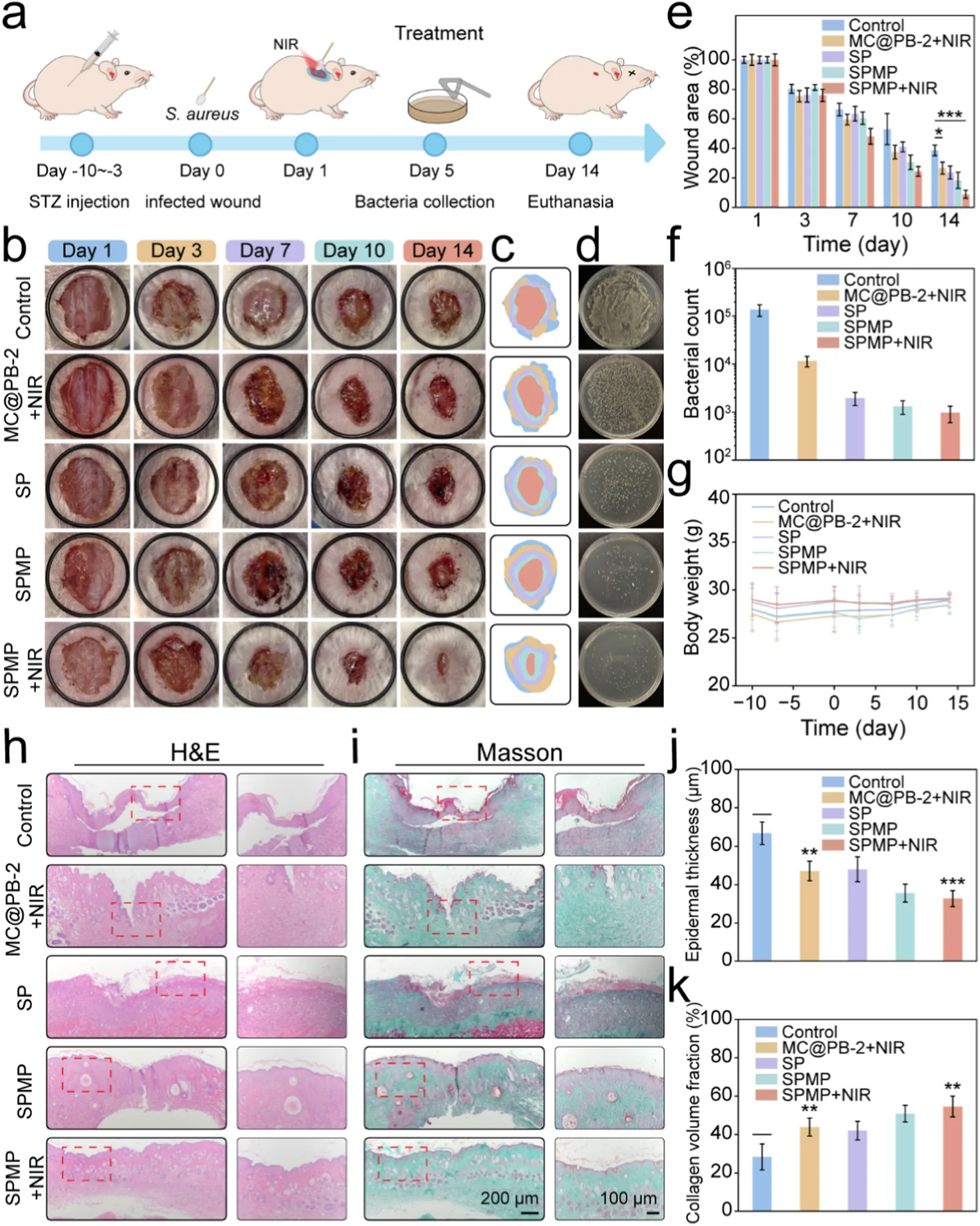
The nanozymes and multifunctional film promote the healing of infected diabetic wounds in mice. (a) Experimental timeline for the modeling process and treatment of infected diabetic wounds in mice. (b) Representative photographs of wounds after different treatments on days 1, 3, 7, 10 and 14. (c) The wound healing trajectory during treatment. (d) Representative photographs of bacterial colonies on day 5. (e) Wound closure rate in different groups (n = 4). (f) Number of *S. aureus* in the collected bacterial suspension (per mL) from wounds on day 5. (g) Weight changes in mice under different treatments. (h) H&E staining and (i) Masson's trichrome staining of wound tissue with different treatments on day 14. (j) Epidermal thickness and (k) collagen volume fraction of wound tissue with different treatments on day 14.

To further investigate the wound healing status across different groups, wound tissue samples were collected from mice in each group on day 14 and subjected to section staining for histological analysis. H&E staining was used to assess epidermal thickness in the wounds of each group. The results indicated that the wounds in the SPMP + NIR group exhibited a thinner epidermis, whereas the control group showed incomplete re-epithelialization (Fig. 7h–j). Masson staining showed that the SPMP + NIR group exhibited the most abundant collagen volume fraction, while the MC@PB-2 + NIR, SP, and SPMP groups also showed greater collagen volume fraction than the control group (Fig. 7i–k).

Furthermore, following treatment with MC@PB-2 + NIR and SPMP + NIR, the expression of IL-6 and TNF-α was significantly reduced compared with the control group (Fig. 8a–d). Quantification showed that IL-6 decreased from 100 ± 11.05% in the control group to 72.91 ± 10.03% and 56.68 ± 7.88% in the MC@PB-2 + NIR and SPMP + NIR groups, respectively (p < 0.01), while TNF-α decreased from 100 ± 9.35% to 68.98 ± 9.57% (p < 0.05) and 49.35 ± 7.52% (p < 0.01). These results indicate a significant alleviation of the pro-inflammatory microenvironment at the wound site, which may be attributed to the reduced bacterial burden and the scavenging of excess ROS. Immunohistochemical staining for the endothelial cell marker CD31 showed a significant increase in angiogenesis on day 14 in both the MC@PB-2 + NIR and SPMP + NIR groups (2.14 ± 0.28-fold and 2.81 ± 0.29-fold vs 1.00 ± 0.20 in the control group, respectively; p < 0.01; Fig. 8e and f), consistent with previous reports that mild photothermal stimulation promotes angiogenesis [36]. A limitation of this study is that the *in vivo* group design did not include MC@PB-2 without NIR or SP + NIR, which would have allowed a full factorial comparison of the independent contributions of MC@PB-2 and NIR. In addition, the ethanol-rich SP/SPMP precursor may contribute to the initial antibacterial effect, which complicates the interpretation of the nanozyme-mediated antibacterial activity. Although the MC@PB-2 + NIR group (without ethanol) showed clear antibacterial effects and the *in vitro* data partially address these issues, further studies using ethanol-free formulations and additional *in vivo* groups are warranted. Collectively, these results demonstrate that SPMP + NIR alleviates inflammatory responsesenhances CD31-positive vascularization, supporting its potential as a wound dressing.

**Fig. 8:**
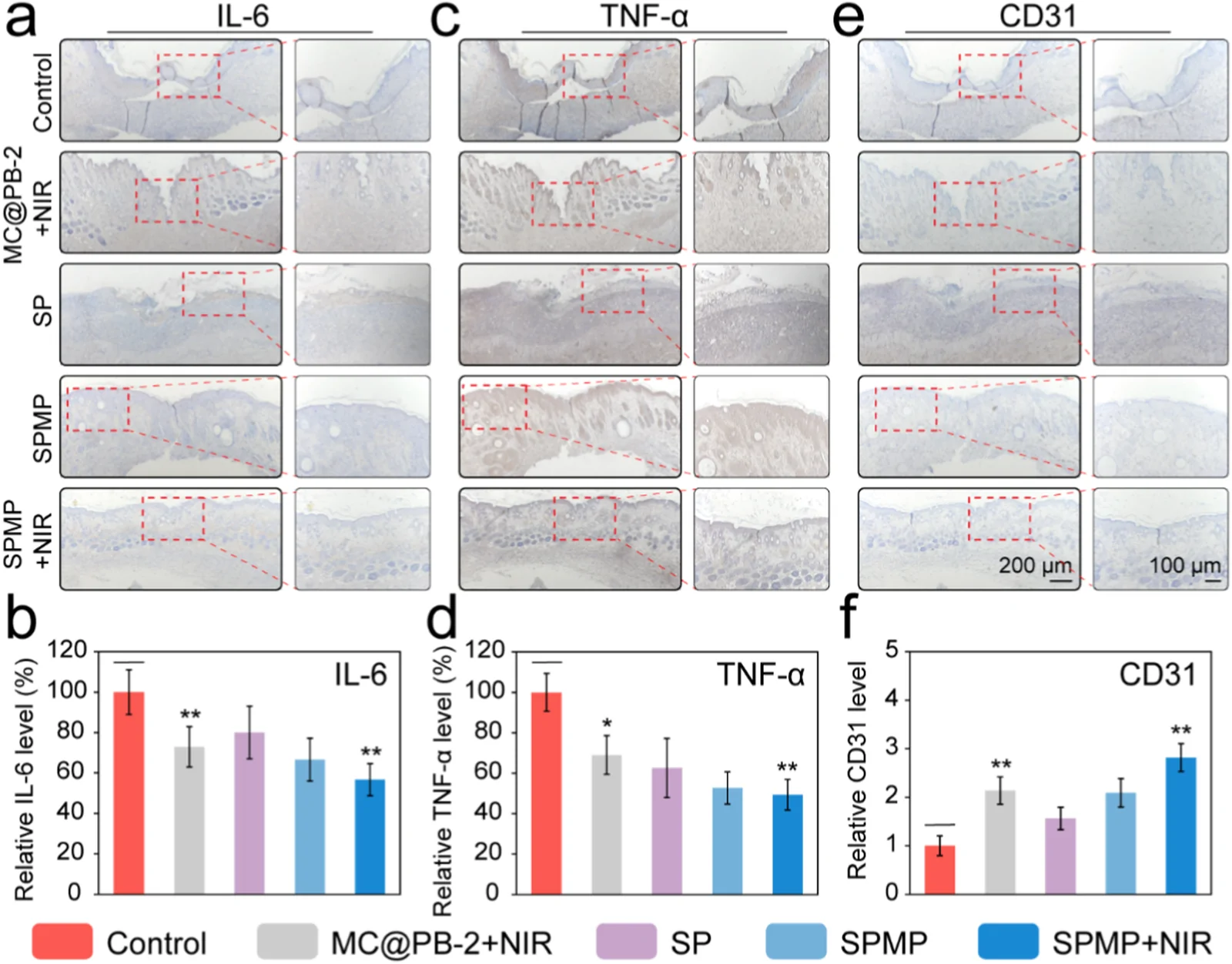
Immunohistochemical analysis of TNF-α, IL-6, and CD31 in wound tissues. Representative images and corresponding quantitative analysis are shown for TNF-α (a, b), IL-6 (c, d), and CD31 (e, f). TNF-α and IL-6 data were normalized to the control group (set as 100%); CD31 data are presented as fold change relative to the control group (set as 1). Data are mean ± SD. *p < 0.05, **p < 0.01.

## 3 Conclusion

In summary, Mn-Cur nanozymes were successfully fabricated at room temperature through sequential coordination and self-assembly of Mn^2+^ with curcumin ligands, followed by surface modification with PB to yield MC@PB nanocomposites. The resulting nanozymes exhibited intrinsic SOD- and CAT-like activities, enabling the efficient scavenging of H_2_O_2_ and •O_2_^−^ through a cascade reaction. Moreover, Mn-Cur effectively scavenged •OH, while PB modification maintained this activity, thereby providing comprehensive antioxidant protection. Additionally, MC@PB demonstrated effective antibacterial activity under NIR irradiation and attenuated pro-inflammatory macrophage activation while promoting an immunoregulatory phenotype, contributing to the alleviation of inflammatory responses. To translate these functions into a practical wound dressing, MC@PB was incorporated into an SF-PVB matrix to form a multifunctional film, denoted SPMP. This film can form in situ on wounds, providing breathability and moisture retention while allowing facile removal. In an infected diabetic wound model in mice, treatment with SPMP under NIR irradiation resulted in reduced bacterial burden, alleviated inflammation, and enhanced angiogenesis, ultimately leading to accelerated wound closure. Collectively, these findings support the potential of SPMP as a therapeutic dressing for infected diabetic wound management.

## 4 Experimental section

### 4.1 Materials

Manganese acetate tetrahydrate (Mn(OAc)_2_·4H_2_O), 2,2′-azino-bis (3-ethylbenzothiazoline-6-sulfonic acid) (ABTS), 2,2-diphenyl-1-picrylhydrazyl (DPPH), riboflavin, isopentyl acetate, thiazolyl blue tetrazolium bromide (MTT), streptozotocin (STZ) were purchased from Aladdin Reagent Co., Ltd. (Shanghai, China). Curcumin, iron(III) chloride (FeCl_3_⋅6H_2_O), potassium ferrocyanide (K_4_Fe(CN)_6_), polyvinyl butyral (PVB), potassium persulfate (K_2_S_2_O_8_), ferrous sulfate (FeSO_4_), salicylic acid (SA), _L_-methionine, nitrotetrazolium blue chloride (NBT), glutaric dialdehyde (25%) were purchased from Macklin Biochemical Co., Ltd. (Shanghai, China). N, N′-dimethylformamide (DMF), ethanol absolute, hydrogen peroxide (H_2_O_2_), sodium carbonate and calcium chloride were purchased from Sinopharm Chemical Reagent Co., Ltd. (Shanghai, China). Agar powder, yeast extract fermentation, tryptone were purchased from Shuangxuan Microbe Culture Medium Products Factory (Beijing, China). Phosphate buffer saline (PBS), DMEM/F-12 (1:1) basic (1×) were purchased from Gibco laboratory. CM-0190 was purchased from Procell (Wuhan, China) ELISA Kits were purchased from Proteintech Group, Inc. (Rosemont, USA). The DAPI, hematoxylin and eosin (H&E) staining kit, Masson staining kit, were purchased from Beyotime (China). Primary antibodies for iNOS, CD31 were purchased from Abcam. (UK).

### 4.2 Synthesis of Mn-Cur and MC@PB

Mn(OAc)_2_·4H_2_O (38 mg) and curcumin (86 mg) were separately dissolved in 2.5 mL of a mixed solvent of DMF and anhydrous ethanol (7:3, v/v). The two solutions were combined in a beaker and magnetically stirred until homogeneous, followed by the addition of 95 mL of the same solvent mixture to induce nanoparticle precipitation. The mixture was aged under stirring at 25 °C for 12 h and then centrifuged at 10,000 rpm for 5 min. The precipitate was collected and washed three times with anhydrous ethanol by centrifugation to obtain Mn-Cur.

Mn-Cur (1 mg/mL) was dispersed in K_4_[Fe(CN)_6_] aqueous solutions at various concentrations (0.25, 0.5, 1, and 2 mM). Subsequently, an equal volume of FeCl_3_ aqueous solution at the same concentration was slowly added dropwise under stirring (500 rpm). After completion of the dropwise addition, the product was washed twice with deionized water to obtain MC@PB. The products prepared at 0.25 and 1 mM were designated as MC@PB-1 and MC@PB-2, respectively.

### 4.3 Preparation of SP and SPMP

Silk fibroin was extracted and the SP solution was prepared according to a previously reported method with minor modifications [11]. The obtained silk fibroin was dissolved in deionized water to obtain a 6% (w/v) solution (designated as L1). Polyvinyl butyral (PVB) was dissolved in anhydrous ethanol to obtain a 20% (w/v) solution (designated as L2). Subsequently, L1 and L2 were mixed at a volume ratio of 1:3 and stirred uniformly to obtain the SP stock solution. For internal structure characterization, 50 μL/cm^2^ of the solution was cast into a mold and allowed to form a film spontaneously.

MC@PB-2 nanozymes were dispersed into L1 by ultrasonication combined with stirring at a concentration of 200 μg/mL. The resulting dispersion was then mixed with L2 at a volume ratio of 1:3 and stirred uniformly to obtain the SPMP stock solution. The film preparation procedure for SPMP was identical to that described for SP.

### 4.4 Antioxidant activity evaluation

#### 4.4.1 •O_2_^−^ scavenging activity

The **•**O_2_^−^ scavenging ability of Mn-Cur, MC@PB-1, and MC@PB-2 was evaluated using the NBT colorimetric method [39]. Briefly, a mixed solution containing NBT (75 μM), L-methionine (13 mM), and riboflavin (20 μM) was incubated for 5 min to generate **•**O_2_^−^. Then the supernatant was incubated with Mn-Cur, MC@PB-1, or MC@PB-2 at various concentrations (0, 50, 100, 150, and 200 μg/mL) for 60 min, respectively. After exposed to LED light for 3 min, the absorbance of the supernatant at 560 nm was measured. The scavenging ratio was calculated using the following formula:

(1) $$\cdot O_{2}^{-}\;\text{scavenging}\;\text{ratio}\;\left(\%\right)=\frac{A_{c}\;‐\;A_{n}}{A_{c}}\times 100$$

where A_c_ and A_n_ represent the absorbance of the control group and the nanozyme-treated groups, respectively.

#### 4.4.2 CAT-like activity

Mn-Cur, MC@PB-1, and MC@PB-2 were separately dispersed in MOPS buffer (pH 7.4) at concentrations of 50, 100, and 200 μg/mL. The dispersions were incubated with H_2_O_2_ (5 mM) at 37 °C, and the dissolved oxygen concentration was monitored in real time using a dissolved oxygen meter.

#### 4.4.3 •OH scavenging activity

The •OH scavenging ability of Mn-Cur, MC@PB-1, and MC@PB-2 was evaluated using a Fenton reaction system (Fe^2+^/H_2_O_2_) with salicylic acid (SA) as a probe. Briefly, H_2_O_2_ (5 mM) and FeSO_4_ (2 mM) were incubated for 10 min to generate •OH. The supernatant was then incubated with Mn-Cur, MC@PB-1, or MC@PB-2 at various concentrations (0, 50, 100, 150 and 200 μg/mL) for 60 min. Subsequently, SA solution (1.5 mM) was added, and the absorbance at 510 nm was measured. The scavenging ratio was calculated using the following formula:

(2) $$\cdot \text{OH}\;\text{scavenging}\;\text{ratio}\;\left(\%\right)=\frac{A_{c}‐A_{n}}{A_{c}}\times 100$$

where A_c_ and A_n_ represent the absorbance at 510 nm of the control group and the nanozyme-treated groups, respectively.

#### 4.4.4 ABTS•^+^ scavenging activity

The activated ABTS•^+^ solution was diluted to adjust the absorbance at 734 nm to approximately 1.0. Subsequently, the ABTS•^+^ solution was incubated with Mn-Cur, MC@PB-1, or MC@PB-2 at various concentrations (0, 50, 100, 150, and 200 μg/mL) in the dark for 60 min. The scavenging ratio was calculated using the following formula

(3) $$\text{ABTS}\cdot +\text{scavenging}\;\text{ratio}\;\left(\%\right)=\frac{A_{c}\;‐\;A_{n}}{A_{c}}\times 100$$

Where A_c_ and A_n_ represent the absorbance at 734 nm of the control group and the nanozyme-treated groups, respectively.

#### 4.4.5 DPPH radical scavenging activity

DPPH• ethanol solution (0.4 mM) was incubated with Mn-Cur, MC@PB-1, or MC@PB-2 at various concentrations (0, 50, 100, 150 and 200 μg/mL) in the dark for 60 min. The scavenging ratio was calculated as follows:

(4) $$\text{DPPH}\cdot \;\text{scavenging}\;\text{ratio}\;\left(\%\right)=\frac{A_{c}\;–\;A_{n}}{A_{c}}\times 100$$

where, A_c_ is the absorbance (516 nm) of the control; A_n_ is the absorbance of groups with nanozymes.

### 4.5 Mechanical properties of SP and SPMP films

The SP and SPMP stock solutions were cast into a 1 × 2 cm mold at 50 μL/cm^2^ and allowed to stand for 2 h for complete film formation. After demolding, tensile testing was performed using a universal testing machine at a stretching rate of 20 mm/min until fracture.

### 4.6 Water vapor transmission and hydrophilicity of SP and SPMP films

Permeability: Deionized water (9 mL) was added to a 10 mL centrifuge tube, and the tube opening was sealed with either an SP or SPMP film. The tubes were then placed in an incubator at 37 °C. At predetermined time intervals, each tube was weighed. The water vapor transmission rate (WVTR) was calculated using the following formula:

(5) $$\text{WVTR}\;\left(\text{mg}\;\text{cm}-2\;h-1\right)=\frac{M_{0}‐M_{t}}{s\times t}$$

where M_0_ is the initial weight (mg), M_t_ is the weight at each time point (mg), s is the area of the tube opening (cm^2^) and t is the incubation time (h).

Hydrophilicity: The hydrophilicity of the SP and SPMP films was evaluated using a static water contact angle measuring instrument equipped with video capture. A 1 μL droplet of deionized water was deposited onto the film surface, and images of the droplets were recorded at 0 and 0.5 h. Three different positions were tested on each film sample.

### 4.7 Antibacterial property of Mn-Cur, MC@PB-1 and MC@PB-2

The antibacterial activity of Mn-Cur, MC@PB-1, and MC@PB-2 against *S. aureus* and *E. coli* was evaluated using the plate colony counting method. Briefly, bacterial suspensions (1 × 10^8^ CFU/mL) were mixed with Mn-Cur, MC@PB-1, or MC@PB-2 (200 μg/mL). The mixtures were then irradiated with 808 nm near-infrared light for 10 min to raise and maintain the temperature at 50 °C. After irradiation, the samples were incubated at 37 °C for 24 h. The bacterial suspensions were then diluted, spread onto LB agar plates, and incubated at 37 °C for 20 h, after which the number of colonies was counted.

To evaluate the concentration-dependent antibacterial activity, the nanozymes were tested at various concentrations (50, 100, and 200 μg/mL) against bacterial suspensions at 1 × 10^8^ CFU/mL. For mechanistic studies, bacteria subjected to different treatments were stained using an AO/EB dual-staining kit and observed under a fluorescence microscope. Bacterial morphology was examined by SEM after dehydration. Additionally, the absorbance of the supernatants at 260 nm was measured using a UV–vis spectrophotometer to assess membrane integrity. The antibacterial ratio was calculated using the following formula:

(6) $$\text{Antibacterial}\;\text{ratio}\;\left(\%\right)=\frac{{\text{CFU}}_{c}\;‐\;{\text{CFU}}_{n}}{{\text{CFU}}_{c}}\times 100$$

where CFUc and CFUn represent the number of colonies in the control group and the nanozyme-treated groups, respectively.

### 4.8 *In vitro* biocompatibility of MC@PB-2 and SPMP

The cytotoxicity of Mn-Cur, MC@PB-1, MC@PB-2, SP, and SPMP was evaluated using the MTT assay. Briefly, HUVECs were seeded into 96-well plates at a density of 1 × 10^4^ cells per well and cultured for 24 h to allow cell attachment. The cells were then treated with various concentrations (50, 100, 150, and 200 μg/mL) of each nanozyme (Mn-Cur, MC@PB-1, MC@PB-2) for 24 h, with untreated cells serving as the control. After incubation, the culture medium was replaced with 9% MTT solution, and the resulting formazan crystals were dissolved in dimethyl sulfoxide (DMSO). The absorbance was recorded at 490 nm using a microplate reader.

For the extract-based cytotoxicity test, SP and SPMP samples (40 mg each) were separately immersed in 5 mL of DMEM-F12 medium and incubated at 37 °C for 24 h to obtain the corresponding extracts. HUVECs were seeded at 2 × 10^3^ cells per well and exposed to the extracts for up to 72 h, with the extracts being refreshed every 24 h. At 24, 48, and 72 h, cell viability was assessed using both the MTT assay and Calcein-AM/PI live/dead staining. The relative cell viability was calculated as follows:

(7) $$\text{Cell}\;\text{viability}\;\left(\%\right)=\frac{A_{n}}{A_{c}}\times 100$$

where A_c_ represents the absorbance of the control group, and A_n_ represents the absorbance of the experimental group (with nanozyme or extract treatment).

The hemolytic activity of Mn-Cur, MC@PB-1, and MC@PB-2 was evaluated using a hemolysis assay. Briefly, fresh bovine blood was centrifuged (3000 rpm, 5 min, 4 °C) and rinsed with PBS (4 °C) for 3 times to obtain red blood cells (RBCs). Then RBCs were incubated with nanozymes (200 μg/mL) at 37 °C for 2 h. The absorbance of supernatants at 540 nm were measured using microplate reader. Hemolysis ratio was calculated using the following formula:

(8) $$\text{Hemolysis}\;\text{ratio}\;\left(\%\right)=\frac{A_{t}\;‐\;A_{n}}{A_{p}‐{\;A}_{n}}\times 100$$

Where A_p_, A_t_ and A_n_ represent the absorbance of the positive control (water), experimental group (nanozyme-treated) and negative group (PBS-treated), respectively.

### 4.9 Anti-inflammation activity of MC@PB-2

RAW 264.7 macrophages were seeded into 96-well plates at a density of 5 × 10^3^ cells per well and cultured for 24 h. The cells were then divided into two groups: a normal control group (untreated) and an LPS-stimulated group, which was treated with lipopolysaccharide (LPS, 100 ng/mL) for 24 h to induce an inflammatory response. After LPS stimulation, the culture medium was replaced with fresh medium containing Mn-Cur, MC@PB-1, or MC@PB-2 (15 μg/mL), or fresh medium without nanozymes (model control group). The normal control group received fresh medium only. After 24 h of further incubation, the levels of interleukin-6 (IL-6), tumor necrosis factor-α (TNF-α), interleukin-10 (IL-10), transforming growth factor-β (TGF-β) and arginase-1 (Arg-1) in the culture supernatants were quantified using enzyme-linked immunosorbent assay (ELISA) kits.

For the measurement of intracellular nitric oxide (NO) and reactive oxygen species (ROS), the same cell modeling and nanozyme treatment procedures were applied. After the corresponding treatments, the levels of NO and ROS were determined using a nitric oxide detection kit and a reactive oxygen species detection kit, respectively.

### 4.10 Cell migration

HUVECs were seeded into 6-well plates at a density of 2.5 × 10^5^ cells per well and cultured in DMEM-F12 medium supplemented with 10% fetal bovine serum (FBS) until reaching confluence. A linear scratch wound was then created across each well using a sterile pipette tip, generating three parallel scratches per well. The detached cells were removed by washing with PBS, and the medium was replaced with DMEM-F12 containing 2% FBS (as the control group) or with the same medium supplemented with MC@PB-2 (50 μg/mL), SP extract, or SPMP extract (as the experimental groups). Photographs of the scratched areas were taken at 0, 12, and 24 h under an inverted microscope. The wound closure rate (migration ratio) was calculated using the following formula:

(9) $$\text{Migration}\;\text{ratio}\;\left(\%\right)=\frac{A_{c}‐A_{n}}{A_{c}}\times 100$$

where A_c_ and A_n_ represent the blank areas of the control and experimental groups, respectively.

### 4.11 *In vivo* wound healing evaluation

All animal experiments were conducted in compliance with the regulations and guidelines approved by the Animal Care and Use Committee of Wuhan University of Technology (Approval No.: WHUT2025-041). The therapeutic efficacy of the MC@PB and films was evaluated using a full-thickness wound defect model in diabetic mice. Kunming mice (male, 6-8 weeks, 20-25 g) were obtained from the Hubei Provincial Center for Disease Control and Prevention. After one week of adaptive feeding, all mice were intraperitoneally injected with streptozotocin (STZ, 50 mg/kg) daily for one week. Three days after the final injection, blood glucose levels were measured using a glucose meter, and mice with levels above 16.7 mmol/L were considered successfully diabetic. Subsequently, a circular full-thickness wound (10 mm in diameter) was created on the back of each mouse, and a bacterial suspension (1 × 10^9^ CFU/mL) was uniformly applied to the wound. After infection for one day, the mice were randomly divided into five groups (n = 4 per group): (1) Control, (2) MC@PB-2 + NIR (100 μg/mL), (3) SP, (4) SPMP, and (5) SPMP + NIR. Following different treatments, wound size and body weight were recorded daily. On day 14, wound skin tissues were collected and fixed in 4% paraformaldehyde (PFA). Hematoxylin and eosin (H&E) staining, Masson's trichrome staining, and immunohistochemical staining (for IL-6, TNF-α, and CD31) were performed for histological analysis. Wound area, epidermal thickness, and collagen deposition were quantified using ImageJ software.

### 4.12 Statistical analysis

All experiments were performed at least three times. Data are expressed as mean ± standard deviation (SD), and statistical comparisons were evaluated using Student's t-test. Significance levels are indicated as *p < 0.05, **p < 0.01, and ***p < 0.001; ns denotes not significant.

## CRediT authorship contribution statement

**Yuxin Wang:** Writing – original draft, Visualization, Validation, Methodology, Investigation, Formal analysis, Data curation, Conceptualization. **Long Wang:** Visualization, Validation, Data curation. **Weifeng Yang:** Funding acquisition, Data curation. **Bingjie Sun:** Methodology, Data curation. **Xinyao Huang:** Methodology, Data curation. **Xiaohong Wang:** Methodology, Investigation. **Meiyu Zhang:** Funding acquisition, Data curation. **Xinyu Wang:** Resources. **Jing Tu:** Writing – review & editing, Supervision, Project administration, Funding acquisition, Conceptualization.

## Declaration of competing interest

The authors declare that they have no known competing financial interests or personal relationships that could have appeared to influence the work reported in this paper.

## Data Availability

Data will be made available on request.
