# Supplementary material for: In situ-forming manganese-curcumin nanozyme film promotes diabetic wound healing via photothermal antibacterial and anti-inflammatory actions
Source: Mater Today Bio. 2026 Oct 19;41:103759. doi: 10.1016/j.mtbio.2026.103759 (PMC13717171; doi:10.1016/j.mtbio.2026.103759)
Supplement: Supplementary file 1 [file mmc1.docx]

**Supporting Information**

**In Situ-Forming Manganese-Curcumin Nanozyme Film Promotes Diabetic Wound Healing via Photothermal Antibacterial and Anti-Inflammatory Actions**

Yuxin Wang ^a^, Long Wang ^a^, Weifeng Yang ^b *^, Bingjie Sun ^a^, Xinyao Huang ^a^, Xiaohong Wang ^a^, Meiyu Zhang ^b *^, Xinyu Wang ^a^, Jing Tu ^a^ ^*^

^a^ State Key Laboratory of Advanced Technology for Material Synthesis and Processing, Wuhan University of Technology, Wuhan 430070, China

^b^ Experimental Research Center, China Academy of Chinese Medical Sciences, Beijing 100700, China

E-mail address: sunzhuyang@126.com; meiyu96@126.com; tujing@whut.edu.cn

*Corresponding author

**Figure S1** Schematic illustration of the dilution and direct mixing methods. (a) Digital images showing the Mn-Cur fabrication process using the dilution method at different time points. (b) TEM images and (c) corresponding particle size distributions of Mn-Cur obtained by the dilution method after 4, 6, and 24 h. (d) Digital images showing the Mn-Cur fabrication process using the direct mixing method at different time points. (e) TEM images and (f) corresponding particle size distributions of Mn-Cur obtained by the direct mixing method at different time points.

**Figure S2** Photographs and SEM images of MC@PB synthesized with FeCl₃ and K₄[Fe(CN)₆] at various equimolar concentrations (1:1 molar ratio): (a) 0.25 mM, (b) 0.5 mM, (c) 1 mM, and (d) 2 mM.

**Figure S3** EDS elemental mapping images showing the spatial distribution of C, O, and Mn in (a) Mn-Cur; and C, O, Mn, Fe, and N in (b) MC@PB-1.

**Figure S4** XPS survey spectra of Mn-Cur and MC@PB-2, with the corresponding atomic percentages of detected elements (C, N, O, Mn, and Fe) listed in the inset table.

**Figure S5** UV–vis absorption spectra of (a–c) NBT photoreduction inhibition indicating superoxide anion (•O₂⁻) scavenging, (d–f) 2-hydroxysalicylic acid (2OH-SA) indicating hydroxyl radical (•OH) scavenging, (g–i) ABTS•⁺ radical scavenging, and (j–l) DPPH radical scavenging after treatment with different concentrations of Mn-Cur, MC@PB-1, and MC@PB-2, respectively.

**Figure S6** Photothermal properties of MC@PB-2. (a) Temperature elevation curves at different concentrations under NIR irradiation (1.5 W/cm²). (b) Temperature elevation curves under NIR irradiation at different power densities (0.5, 1.0, 1.5, and 2.0 W/cm²) at a concentration of 100 μg/mL. (c) Photothermal stability curves over four cycles of NIR irradiation (1.5 W/cm²) at a concentration of 100 μg/mL. (d) Linear fit of time versus-ln θ from the cooling period for calculating the photothermal conversion efficiency.

**Figure S7** Photographs of (a) *S. aureus* and (b) *E. coli* colonies on agar plates after incubation with different concentrations of Mn-Cur, MC@PB-1, and MC@PB-2 for 24 h without NIR irradiation.

**Figure S8** (a) Live/Dead staining images of HUVECs after treatment with MC@PB-2 (50 μg/mL) and extracts (SP and SPMP) for 24, 48, and 72 h. (b) Viability of HUVECs treated with extracts (SP and SPMP).
